# Predictors for Pulmonary Tuberculosis Treatment Outcome in Denmark 2009–2014

**DOI:** 10.1038/s41598-019-49439-9

**Published:** 2019-09-10

**Authors:** Inge K. Holden, Troels Lillebaek, Niels Seersholm, Peter H. Andersen, Christian Wejse, Isik S. Johansen

**Affiliations:** 1 Department of Infectious Diseases, Odense University Hospital, Odense, Denmark; 2 Department of Clinical Research, University of Southern Denmark, Odense, Denmark; 3 Mycobacterial Centre for Research Southern Denmark – MyCRESD, Odense, Denmark; 4 International Reference Laboratory of Mycobacteriology, Statens Serum Institut, Copenhagen, Denmark; 5 Department of Internal Medicine, Herlev and Gentofte University Hospital, Hellerup, Denmark; 6 Department of Infectious Disease Epidemiology and Prevention, Statens Serum Institut, Copenhagen, Denmark; 7 Department of Infectious Diseases, Aarhus University Hospital, Aarhus, Denmark

**Keywords:** Infectious diseases, Tuberculosis

## Abstract

Monitoring of tuberculosis (TB) treatment outcome is essential to ensure an effective TB control program. In this nationwide retrospective cohort study from Denmark we present TB treatment outcome rates and risk factors associated with an unfavourable outcome. All patients notified with pulmonary TB from 2009 through 2014 were included. Logistic regression analyses were used to identify risk factors for unfavourable outcome. In total, 1681 pulmonary TB cases were included. TB treatment success rates increased during the study period. In 2014, the treatment success rate reached 85% for new culture positive cases whereas 7% cases interrupted treatment. The mortality decreased during the study period from 12.3% to 4.1%. Several risk factors associated with unfavourable outcome were identified in a multivariable model: male (OR: 2.56), Greenlandic origin (OR: 1.80), abuse of alcohol (OR: 2.90), history of mental disorder (OR: 2.46), and anaemia at time of treatment initiation (OR: 1.92). In a TB low incidence setting such as the Danish, it is important to maintain focus on preventing an unfavourable TB outcome. Patient management and treatment can be optimized by taking into consideration risk factors such as those identified in the present study.

## Introduction

Timely and effective tuberculosis (TB) treatment is a cornerstone in TB control in order to cure the patient, limit the transmission and prevent drug resistance^1–3^. Thus, TB treatment outcome monitoring is essential when evaluating TB control strategies. Furthermore, treatment outcome monitoring allows comparison between European countries and geographic areas. Denmark has a low incidence of TB. In 2017, 275 TB cases were reported to the Danish national TB surveillance system resulting in an incidence of 4.8/100.000 population. The incidence trend has been decreasing since 2001 (9.6/100.000 population).

In Denmark, reporting of TB treatment outcome is voluntary. As a result, the outcome data are incomplete and often reported with delay. TB treatment outcome data from 2008 through 2010 was published in 2013 and revealed a success rate for new culture positive pulmonary TB (PTB) cases of 79%^4^. In the most recent Surveillance Report from the European Centre for Disease Prevention and Control (ECDC), Denmark reported a treatment success rate 37.5% in 2017, however 45.7% of the cases had not been evaluated^5^. This suggests the true treatment success rate was higher.

For countries within the European Union and European Economic Area, the World Health Organization (WHO) and ECDC recommend a treatment success rate of 85% at 12 months for all new culture positive PTB cases and a treatment success rate of 90% for all new smear positive PTB cases^6,7^. This indicates that Denmark has not been able to meet WHO/ECDC targets for TB treatment success.

Identifying and targeting risk factors for an unfavourable outcome is potentially beneficial in order to improve treatment success rates. Ideally, the probability of an unfavourable outcome should be estimated from information available at the time of TB diagnosis and taken into account.

This study was conducted in order to provide updated and complete data on treatment outcome of PTB in Denmark. Furthermore, to explore predictors for an unfavourable treatment outcome in order to ensure better treatment success rates in the future.

## Methods

We conducted a nationwide retrospective cohort study including all patients notified with PTB in Denmark from January 1^st^, 2009 to December 31^st^, 2014.

### Data source

#### Notification data

TB notification has been mandatory in Denmark since 1905 and centralized since 1920^8^. Today, TB is notifiable to the Regional Unit for Supervision and Guidance under the Danish Patient Safety Authority and to the Danish national TB surveillance system hosted by the Department of Infectious Disease Epidemiology & Prevention (DIDEP) at Statens Serum Institut (SSI). The duty of notification applies to the physician responsible for the treatment. TB cases are notified according to WHO definitions^9^. Standardized voluntary reporting of TB treatment outcome was initiated in 2000 in Denmark. The DIDEP provided the following regarding all TB cases notified during the study period: identification codes (Civil Registration numbers – CRN), demographics, immigrant status and date of notification. Status as immigrant was defined as patients born abroad or those born in Denmark for whom one or both parents had been born abroad.

#### Microbiological data

In Denmark, all culture-based analyses are performed at the International Reference Laboratory of Mycobacteriology (IRLM) at SSI. Results of microscopy, Polymerase Chain Reaction (PCR), culture, and drug susceptibility test were obtained from IRLM.

### Danish national patient registry

The Danish National Patient Registry (DNPR) contains data on all admissions to Danish public hospitals since 1977 and data on outpatient contacts since 1994^10^. Data was obtained on all patients, who were notified with TB in Denmark during the study period. Data from DNPR was used to determine history of previous illnesses, including previous mental disorder (ICD10: DF00-DF99). and to calculate Charlson Comorbidity Index (CCI)^11^.

### Data linkage

In order to cross-link the registers, we used the unique Danish CRN, which is assigned to all residents of Denmark at birth or after residing legally in Denmark for 3 months. Patients who do not meet the criteria for obtaining a CRN are assigned a temporary CRN at first point of contact with the healthcare system. To accommodate modification in temporary CRN, probabilistic linkage was done. All CRN obtained from the notification system were linked to the DNPR data and to the data from IRLM.

#### Hospital records

For all patients identified by the notification system, medical records were reviewed for socio-demographics, clinical characteristics, TB treatment, and treatment outcome (Tables 1–3).

**Table 1:** Tuberculosis treatment outcome categories modified from WHO definitions^9^.

Treatment outcome	Definition
Cured	TB confirmed by culture at the beginning of treatment and culture negative in the last month of treatment and on at least one previous occasion.
Treatment Completed	TB treatment completed without evidence of failure, but without fulfilling the above mention criteria
Died	A TB patient who dies for any reason before starting or during TB treatment.
Treatment failed	Positive culture during last month of the continuation phase
Treatment interrupted	TB treatment interrupted for a minimum of 2 consecutive months.
Transfer	A TB patient who permanently leaves Denmark during TB treatment
Not evaluated	A TB patient who does not fit into other categories
Still on treatment	A TB patient who were still on treatment at time of study termination
Treatment success	The sum of cured and treatment completed
Unfavourable treatment outcome	The sum of treatment failed, treatment interrupted and not evaluated

**Table 2:** Socio-demographics of cases with pulmonary tuberculosis in Denmark; 2009–2014.

Characteristics	Danes			Greenlanders			Immigrants			p-value
	n	N	%	n	N	%	n	N	%	
Patients		637	37.9		342	20.3		702	41.8	
**Sex**										
Male	441	637	69.2	195	342	57.0	446	702	63.5	<0.01
**Age**										
(Median, N, IQR)	51	637	40–60	47	342	41–53	34	702	26–45	<0.01
0–24	61	637	9.6	19	342	5.6	158	702	22.5	
25–44	151	637	23.7	116	342	33.9	350	702	49.9	
45–64	309	637	48.5	199	342	58.2	144	702	20.5	
≥65	116	637	18.2	8	342	2.3	50	702	7.1	
**Predisposing factors**										
Alcohol^αα^	295	621	47.5	277	334	82.9	94	660	14.2	<0.01
Tobacco	438	628	69.8	309	334	92.5	287	657	43.7	<0.01
Cannabis	104	598	17.4	191	395	62.6	62	645	9.6	<0.01
History of Illegal drug use	82	634	12.9	21	338	6.2	52	692	7.5	<0.01
Homelessness	5.8	635	5.8	107	340	31.5	60	693	8.7	<0.01
History of incarceration	17	635	2.7	1	340	0.3	18	695	2.6	0.03
Previous TB	70	637	11.0	100	342	29.2	95	640	14.8	<0.01
HIV Positive	12	407	3.0	2	238	0.8	39	497	7.9	<0.01
**Charlson Comorbidity Index**										
0	353	637	55.4	241	342	70.5	479	637	44.6	<0.01
1	133	637	20.9	56	342	16.4	71	637	11.2	<0.01
≥2	151	637	23.7	45	342	13.2	87	637	13.7	<0.01
**Concomitant site**										
Any	27	637	4.2	9	342	2.6	77	702	11.0	<0.01
**Diagnosed supported by**										
Culture Positive	544	621	87.6	310	341	90.9	570	673	84.7	0.02
Smear Positive	368	621	59.3	180	341	52.8	344	673	51.1	0.01
NAA^β^ positive	353	778	45.4	176	220	80.0	363	493	73.6	0.13
Tuberculin skin test	3	35	8.6	2	8	25.0	11	64	17.2	0.46
IGRA^χ^	137	181	75.7	78	82	95.1	234	262	89.3	<0.01

^α^Alcohol abuse was quantified according to the Danish Health Authorities recommendations (more than 14 units pr. week of alcohol for women and more than 21 units for men).

^β^Nucleic amplification acid.

^χ^Interferon Gamma Release Assay.

**Table 3:** Clinical characteristics in pulmonary tuberculosis in Denmark; 2009–2014.

Characteristics	Danes			Greenlanders			Immigrants			p-value
	n	N	%	n	N	%	n	N	%	
Identification by Contact Tracing	96	635	15.1	68	341	19.9	65	697	9.3	<0.01
Symptom Duration^α^ (days) (Median, N, IQR)	60	487	21–120	52.5	220	14–90	60	567	21–90	<0.01
Time to Diagnosis (days) (Median, N, IQR)	9	610	2–33	3	323	0–19	5	664	1–21	<0.01
Weight Loss	337	617	54.6	197	322	61.2	401	671	59.8	0.08
Night Sweat	215	581	37.0	115	297	38.7	281	626	44.9	0.02
Fever	241	626	38.5	110	330	33.3	337	678	49.7	<0.01
Cough	516	632	81.7	279	334	83.5	569	687	82.8	0.74
Hemoptysis	70	626	11.2	53	329	16.1	156	683	22.8	<0.01
Chest Pain	132	622	21.2	70	322	21.7	213	680	31.3	<0.01
Non-adherent	102	619	16.5	158	324	48.8	98	645	15.2	<0.01

^α^Time from symptom onset until first hospital contact.

**Figure 1:**
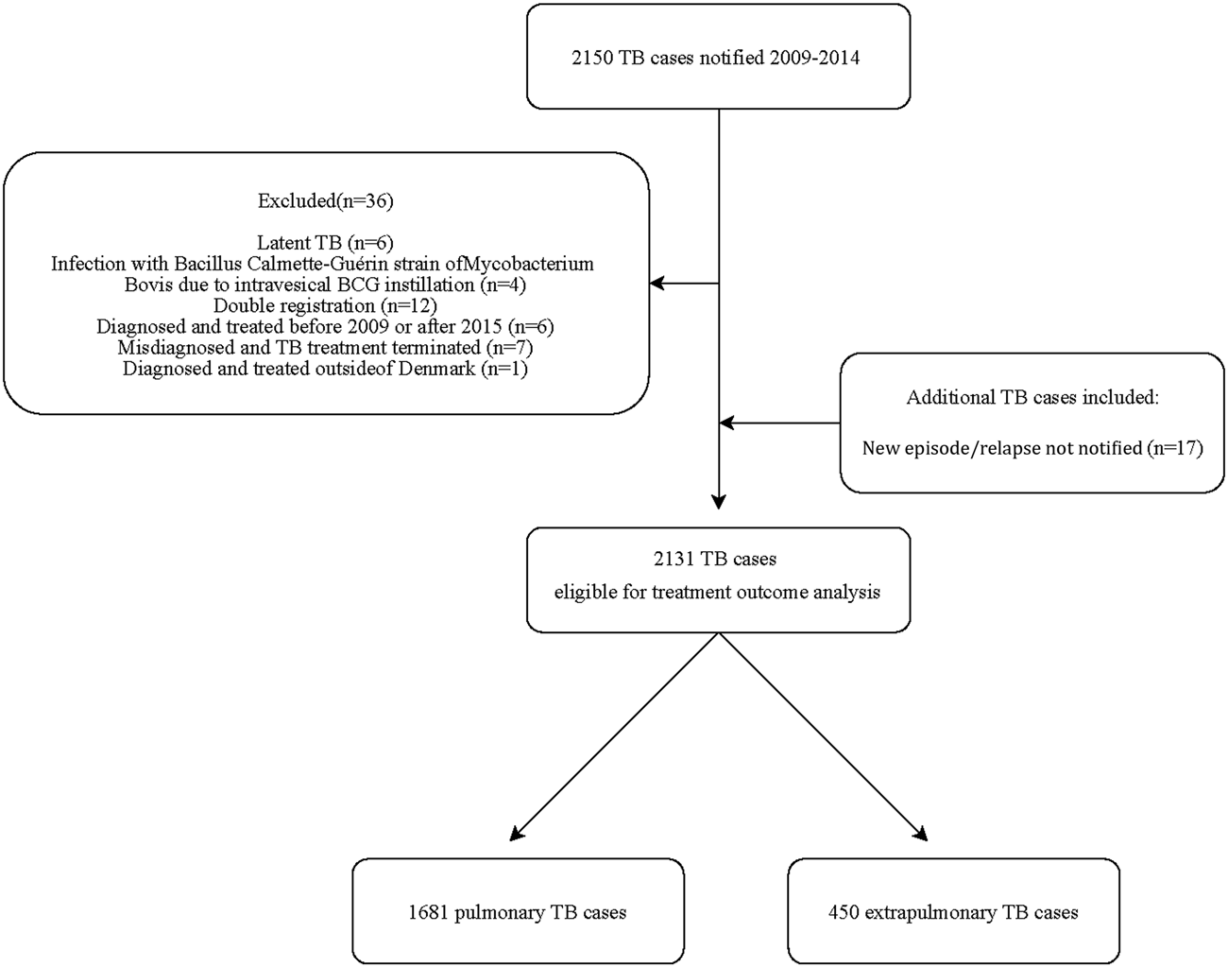
Study population. ^*^A new episode/relapse was defined according to WHO/ECDC guidelines and cases were only included once during a 12 months period.

The study population is described in Fig. 1.

Patients were followed from time of first contact with the hospital due to TB until 1 year after TB treatment was completed.

### Statistical analysis

All PTB cases were included in the data analysis describing patient characteristics. Categorical data was described by total and percentages, the denominator for calculated percentages was the number of cases with known information. Data comparisons were made using the chi-square test or Fisher’s exact test if 20% of expected cell values were ≤5. Continuous variables were described as medians and interquartile ranges and compared using the Wilcoxon rank sum test. A p-value of less than 0.05 (5%) was considered statistically significant.

Extensively drug-resistant (XDR), multidrug-resistant (MDR), isoniazid resistant PTB treated with second-line drugs, and patients who died or transferred out were excluded in the data analysis describing predictors for unfavourable TOs. Cases were excluded if the patient died or transferred from Denmark during the observation period to ensure that all patients in the study population had a chance to complete treatment successfully. Treatment outcome was categorized into two categories as summarized in the last two rows in Table 1.

Characteristics of cases with an unfavourable outcome were compared with cases with the outcome “treatment success”, using univariable logistic regression.

Variables for multivariable analysis were partly selected a priori (country of origin, previous TB). Other variables were selected if they showed a univariable association with defaulting (p-value < 0.05). Wald tests were used to select statistically significant variables for unsuccessful treatment outcome, and the goodness of fit of the model was tested with the Hosmer-Lemeshow method.

Data from hospital records were entered into a Microsoft Excel 2010 (Microsoft, USA) workbook. All statistical analysis was performed using Stata, version 15.2 (StataCorp Inc., USA).

### Ethics

The study was approved by the Danish Data Protection Agency (Jnr. 15/34961) and the Danish Health Authority (Jnr 3-3013-1213/1).

In accordance with Danish law, observational studies performed in Denmark do not need approval from the Medical Ethics Committee or written consent from participants. All analyses are presented anonymously.

## Results

### Population

During the study period, a total of 2,150 TB cases were notified of these 2,131 cases were available for treatment outcome analysis of which PTB accounted for 1681 (78.9%) cases (Fig. 1).

Among the pulmonary cases; previously treated cases represented 16.4% (n = 265). A total of 892 (54.6%) cases were smear positive and 1,424 (89.1%) cases were culture positive. Drug susceptibility testing increased during the study period from 97.6% to 99.1%. Drug resistance was detected in 81 cases (6.0%), of which 1.2% (n = 1) and 9.9% (n = 8) were MDR and XDR TB cases, respectively. The remaining were mainly isoniazid monoresistant.

### Treatment

In total, 1,658 PTB cases started TB treatment, the remaining 23 patients (1.4%) died before treatment was initiated. The median treatment duration was 6 months (IQR: 5.7–6.5).

A total of 819 (50.3%) of drug susceptible PTB cases received the standard treatment of 2 (3) months (the national guideline was modified in 2010: the intensive phase was decreased from 3 to 2 months) intensive phase including 4 drugs (rifampicin, isoniazid, pyrazinamide, ethambutol) and 4 months continuation phase including 2 drugs (rifampicin, isoniazid).

### Socio-demographic and clinical characteristics

The population was subdivided into 3 categories according to country of origin (Table 2). The majority of immigrants originated from Asia (40.0%), with patients from Afghanistan, Pakistan and Philippines accounting for 38.1% cases. Patients from other European countries comprised 29.5%, whereas 28.9% were from Africa among whom the majority originated from Somalia (54.2% of the African cases).

Men accounted for 64.4% and the median age of the study population was 44 years (IQR: 31–54). Abuse of alcohol, tobacco, cannabis, illegal drugs, being homeless, and a history of incarceration was significantly more frequent among men. Individuals who abused alcohol more frequently had one or more of the following risk factors: use of cannabis or other illegal drugs, homelessness (75.6%). While individuals diagnosed with a mental health disorder more frequently had an abuse of illegal drugs or alcohol or were homeless (66.4% vs 49.8%, p < 0.01). A total of 779 (53.7%) had anaemia at time of diagnosis, which was significantly more frequent among women (57.6% vs 51.6%, p = 0.03). The highest proportion of anaemia was among individual from Greenland (64.0%), followed by individuals originating from Africa (59.0) while Danes (47.4%) had the lowest prevalence of anaemia. In cases with anemia there were a significantly greater proportion of smear positive (63.5% vs 45.5%, p < 0.01) and nucleic amplification acid positive (81.3% vs 72.0%, p. < 0.01) sputum samples. In addition, the proportion of sputum samples quantified as “3+ acid fast bacilli” were significantly greater among cases with anemia (42.3% vs 24.1%, p < 0.01).

A concomitant site of TB was significantly more frequent among immigrants (11.0%) with lymphatic TB (29.2%) being the most common site.

A total of 53 (4.6%) was co-infected with HIV with the highest number among immigrants (7.9%). During the study period, the number of cases tested for HIV increased significantly from 49.0% in 2009 to 79.3% in 2014.

### Treatment outcome

**Figure 2:**
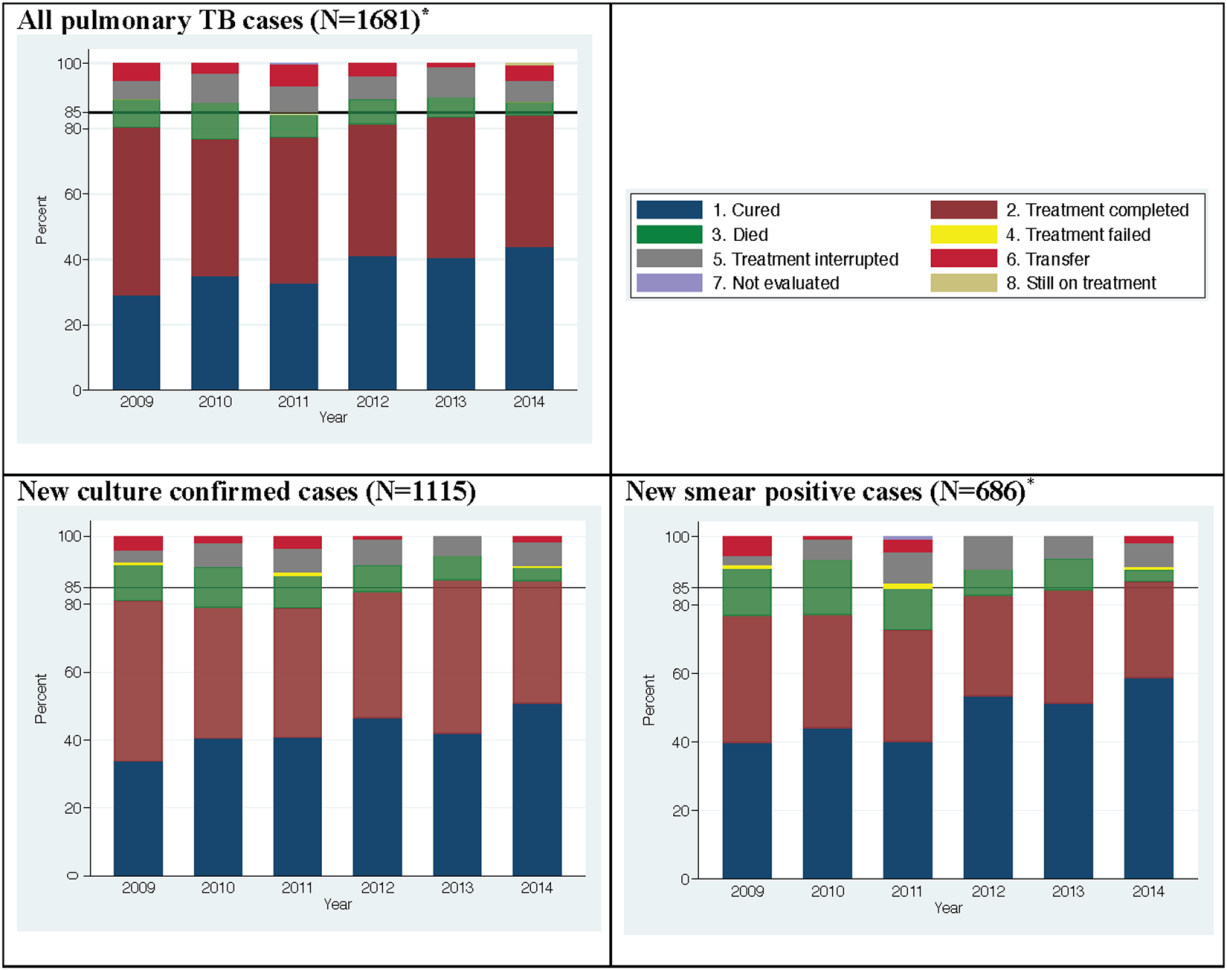
Pulmonary TB treatment outcome in Denmark; 2009–2014. ^*^XDR, MDR and isoniazid resistance cases treated with second-line drugs excluded.

Overall, the proportion classified as treatment success was 80.5% (n = 1353), this increased during the study period from 76.8% to 84.1% with the highest treatment success rate in 2014 (Fig. 2).

The proportion of Greenlanders achieving treatment success was significantly lower (p-value < 0.01) but increased during the study period from 71.1% to 77.8%.

In further analyses the following cases are excluded: XDR-TB (n = 1), MDR-TB (n = 8) and isoniazid resistance cases treated with second-line drugs (n = 41).

Treatment outcome in new culture confirmed and new smear positive cases are presented in Fig. 2. In the univariate analysis male gender, Greenlandic origin, abuse of alcohol, tobacco, cannabis, illegal drugs, homelessness, a history of imprisonment, anaemia, previous TB and mental disorder were associated with an unfavourable outcome (Table 4).

**Table 4:** Odds ratio (OR) for unfavourable outcome vs. treatment success among pulmonary tuberculosis patients in Denmark; 2009–2014.

Characteristics at time of TB diagnosis	Number of cases		Factors associated with unfavourable treatment outcome, univariate logistic regression		Factors associated with unfavourable treatment outcome, multivariate logistic regression^α^	
	Unfavourable outcome (%)	Treatment success (%)	OR	[95% CI]	OR	[95% CI]
**Total**	127 (8.8)	1313 (91.2)				
**Sex**						
Female	28 (5.3)	498 (94.7)	Reference		Reference	
Male	99 (10.8)	815 (89.2)	2.16	1.38-3-38	2.56	1.19–3.63
**Age (years)**						
0–24	14 (6.4)	204 (93.6)	Reference			
25–44	54 (10.2)	478 (89.8)	1.65	0.89–3.04		
45–64	53 (9.5)	505 (90.5)	1.53	0.83–2.82		
≥65	6 (4.5)	126 (95.5)	0.69	0.23–2.06		
**Predisposing factors**						
Alcohol^β^	83 (14.9)	473 (85.1)	3.42	2.29–5.10	2.90	1.67–5.04
Tobacco	109 (12.4)	772 (87.6)	4.44	2.52–7.81		
Cannabis	59 (19.3)	246 (80.7)	4.10	2.75–6.02		
History of Illegal drug use	25 (20.0)	100 (80.0)	2.98	1.79–4.95		
Homelessness	43 (25.3)	127 (74.7)	4.76	3.15–7.19		
History of incarceration	9 (36.0)	16 (64.0)	6.16	2.66–14.31		
Previous TB	33 (14.5)	194 (85.5)	2.17	1.38–3.43	1.47	0.84–2.58
History of mental disorder	17 (16.2)	88 (83.8)	2.15	1.24–3.74	2.46	1.28–4.72
**Charlson comorbidity score**						
0	37 (8.3)	410 (91.7)	0.97	0.64–1.47		
1	19 (8.5)	205 (91.5)	1.00	0.60–1.68		
≥2	18 (8.1)	205 (91.9)	0.94	0.55–1.63		
**Concomitant site**						
Any	2 (2.2)	89 (97.8)	0.22	0.05–0.90		
**Diagnosed supported by**						
Positive Culture	112 (9.3)	1087 (90.7)	1.49	0.83–2.69		
**Country of origin**						
Denmark	40 (7.4)	504 (92.6)	Reference		Reference	
Greenland	46 (15.6)	249 (84.4)	2.33	1.47–3.68	1.80	1.06–3.07
Other	41 (6.8)	560 (93.2)	0.92	0.58–1.47	0.86	0.45–1.64
**Identification by Contact Tracing**	14 (6.5)	203 (93.5)	0.68	0.38–1.21		
**Clinical symptoms**						
Fever, weight loss and cough >3 weeks	33 (9.5)	314 (90.5)	1.1	0.74–1.66		
**Lab results**						
**Hgb (mmol/L)**						
Normal	37 (6.1)	567 (93.9)	Reference		Reference	
Anemia^χ^	67 (10.5)	573 (89.5)	1.79	1.18–2.72	1.92	1.22–3.03
**Treatment**						
Second line	11 (11.5)	85 (88.5)	1.38	0.72–2.65		
INH monoresistance treated with standard regime	5 (21.7)	18 (78.3)	2.78	1.01–7.69		
Adherence^δ^	17 (1.5)	1081 (98.5)	Reference			
Non-adherence	105 (32.2)	221 (67.8)	30.21	17.82–51.22		

^α^1186 case were included in the multivariable analysis. Variables for multivariable analysis were partly selected a priori (country of origin, previous TB). Other variables were selected if they showed a univariable association with defaulting (p-value < 0.05). Wald tests were used to select statistically significant variables for unsuccessful treatment outcome, and the goodness of fit of the model was tested with the Hosmer-Lemeshow method.

^β^Alcohol abuse was quantified according to the Danish Health Authorities recommendations (more than 14 units pr. week of alcohol for women and more than 21 units for men).

^χ^Women: Hgb < 7.5 mmol/L. Men: Hgb < 8.0 mmol/L.

^δ^Non-adherence: if described in patient records and/or ≥ two episodes of non-attendance for clinical appointments.

Standard Error adjusted for clusters in CPR.

The three most frequent symptoms: cough, fever and weight loss were combined with a symptom duration of more than 3 weeks, however there was no association with outcome.

Table 4 shows the final multivariable model fitted on 1,188 cases. A significantly increased risk of an unfavourable outcome was found among males (OR: 2.56; CI: 1.19–3.63), with cases originating from Greenland (OR: 1.80; CI: 1.06–3.07), had alcohol abuse (OR: 2.90; CI: 1.67–5.04), a history of mental disorder (OR: 2.46; CI: 1.28–4.72) or who had anaemia at time of treatment initiation (OR: 1.92; CI: 1.22–3.03).

## Discussion

In this nationwide study, Denmark met the WHO/ECDC recommendations regarding treatment success rate of at least 85% for new culture confirmed PTB cases in 2013 and 2014. An unfavourable outcome was associated with male gender, Greenlandic origin, abuse of alcohol, history of mental disorder, and anaemia at time of diagnosis. The majority of unfavourable outcome was lost to follow up.

The overall treatment success rate for PTB in Denmark was 84% in 2014. In new culture-positive cases, 86.6% were classified as treatment success, of these 51.7% achieved the outcome “cured”. For the cases classified as “treatment completed” (34.9%), a second sputum sample was not obtained during the last month of treatment, as observed in another resent European study^12^. In the End TB Strategy, WHO recommends that the treatment success rate should be at least 90% for drug-susceptible and drug-resistant TB combined by 2025^13^. This requires further improvement in TB surveillance, treatment and control programs in Denmark.

The present voluntary reporting of TB treatment outcome in Denmark resulted in a response rate of only 40% in 2000. In order to improve the response rate, a new procedure was implemented: DIDEP systematically contacted departments with TB patients urging them to report the treatment outcome result. This led to an improved response rate of more than 80% the subsequent years. This indicates that reporting of TB treatment outcome needs to be mandatory in order to achieve completeness of the surveillance system and avoid systematic errors, e.g. not reporting unfavourable outcomes.

Immigrants from TB high burden countries constitute a considerable proportion of TB cases in many European countries^14–19^. In our study, Greenlanders represented about one third of immigrants, thus we subdivided the population into Danes, Greenlanders and Immigrants. We found great disparities between the three groups in terms of age, predisposing factors and adherence to the treatment regime. The population of Greenlanders in Denmark represents 1% of the population but represented 20.3% of the pulmonary TB cases. An earlier study has identified an ongoing spreading of the outbreak strain C2/1112-15 among Greenlanders living in Denmark, this is also in accordance with 20% of the cases were identified by contact tracing^20^. Cases from Greenland had a higher risk of unfavourable outcome which might be attributed to the vast majority (89%) had abuse of alcohol or illegal drugs or were homeless. Immigrants from other countries did not differ significantly from Danes in terms of outcome. This is in discrepancy with previous studies and could be explained by foreign origin being related to migration and thereby to the treatment outcome: “transfer”, which has been included in unfavourable treatment outcome in other studies^14–17,21^. Additionally, only 26% of immigrants had the following risk factors: alcohol or illegal drug use or homelessness.

We identified male gender to be independently associated with an unfavourable outcome. The poorer treatment outcome in men can be attributed to some behavioural components such as alcohol and drug abuse, which we found to be significantly more common among men in this study in line with other EU countries^14–22^.

Increasing age and comorbidity in terms of CCI were not associated with unfavourable outcome. This may be explained as the outcome “died” was excluded in the final analysis. It is well known that mortality from TB increases with increasing age, which will contribute to an unfavourable outcome when the outcome “died” is included^15,16,19,21,23^. However, a history of mental disorder was associated with an unfavourable outcome. It is recognized that mental disorder is associated with poor adherence to medical treatment and earlier studies have also found that mental disorder was associated with non-adherence to TB treatment^24,25^.

Anaemia has been associated with delayed sputum smear conversion among sputum-positive TB patients, advanced disease and mortality^26–29^. Anaemia was associated with an unfavourable outcome in this study. This suggests that anaemia can be a proxy for poor health status and advanced disease, which is also supported by the fact that the bacilli load was greater in this group.

In our study we have identified additional risk factors associated with an unfavorable outcome. In addition, the vast majority of patients categorized with an unfavorable outcome were lost to follow-up (95.4%). These patients could benefit from increased support, monitoring and potentially directly observed therapy (DOT). An increased focus on optimizing the living conditions among homeless, provide support to overcome alcohol/illegal drug use and focus on involving family or other support persons in TB treatment could help to reduce the risk of being lost to follow up.

The limitations of this study are: the population was identified by notification data, hence patients who are not notified were not included. However, a recent study from Denmark has assessed the underreporting of TB to 7.5%^30^. The non-notified cases were all culture-negative and did not differ significantly in treatment outcome and risk factors from the notified cases^30^. All clinical information was from hospital records, hence no direct patient contact. In 24.2% of cases, information regarding symptom duration was missing, potentially underestimating the effect of symptom duration on treatment outcome. CCI could not be assessed for patients with temporary CRN, because they are not registered in the DNPR, these patients accounted for 3.9% (n = 65) of the entire population.

Denmark has the best prospects of obtaining high treatment success rates with state-of-the-art TB diagnostics and free tax-supported medical care. The overall trend of TB incidence has been decreasing since 2001 and the treatment success rates increased during our study period. The decline in incidence has been observed in all three subgroups, with the highest decline among Danish born men. These results are encouraging in terms of the National TB control program. However, Finland and Sweden have observed a more rapid decline in TB incidence among their native populations^31^. More than 6% of cases in Denmark interrupted TB treatment, this group of patients potentially pose a risk of disease progression at the individual level leading to continuous transmission in the society. The Danish public health legislation does not allow the use of mandatory treatment and hospitalization for infectious patients. Whereas there is a provision for DOT, administered at the patient’s home/shelter or at the hospital.

We conclude that the TB treatment success rate has been increasing in Denmark during the study period, reaching the WHO target for new culture confirmed PTB in the last part of the period.

To further reduce unfavourable outcome, risk factors identified by this study should be utilized and preventive approaches should be initiated in order to optimize management of patients, when TB treatment is initiated.
